# Respiratory Health Effects of In Vivo Sub-Chronic Diesel and Biodiesel Exhaust Exposure

**DOI:** 10.3390/ijms24065130

**Published:** 2023-03-07

**Authors:** Katherine R. Landwehr, Ryan Mead-Hunter, Rebecca A. O’Leary, Anthony Kicic, Benjamin J. Mullins, Alexander N. Larcombe

**Affiliations:** 1 Occupation, Environment and Safety, School of Population Health, Curtin University, Perth, WA 6845, Australia; 2 Respiratory Environmental Health, Wal-yan Respiratory Research Centre, Telethon Kids Institute, Perth Children’s Hospital, Nedlands, Perth, WA 6009, Australia; 3 Department of Primary Industries and Regional Development, Perth, WA 6151, Australia; 4 Department of Respiratory and Sleep Medicine, Perth Children’s Hospital, Nedlands, Perth, WA 6009, Australia; 5 Centre for Cell Therapy and Regenerative Medicine, The University of Western Australia, Perth, WA 6009, Australia

**Keywords:** biodiesel, diesel, exhaust exposure, in vivo exposure model, health impact of exhaust exposure, respiratory health

## Abstract

Biodiesel, which can be made from a variety of natural oils, is currently promoted as a sustainable, healthier replacement for commercial mineral diesel despite little experimental data supporting this. The aim of our research was to investigate the health impacts of exposure to exhaust generated by the combustion of diesel and two different biodiesels. Male BALB/c mice (*n* = 24 per group) were exposed for 2 h/day for 8 days to diluted exhaust from a diesel engine running on ultra-low sulfur diesel (ULSD) or Tallow or Canola biodiesel, with room air exposures used as control. A variety of respiratory-related end-point measurements were assessed, including lung function, responsiveness to methacholine, airway inflammation and cytokine response, and airway morphometry. Exposure to Tallow biodiesel exhaust resulted in the most significant health impacts compared to Air controls, including increased airway hyperresponsiveness and airway inflammation. In contrast, exposure to Canola biodiesel exhaust resulted in fewer negative health effects. Exposure to ULSD resulted in health impacts between those of the two biodiesels. The health effects of biodiesel exhaust exposure vary depending on the feedstock used to make the fuel.

## 1. Introduction

Diesel exhaust exposure is known to lead to negative health impacts on multiple organ systems including, but not limited to, the respiratory, cardiovascular, nervous, endocrine, and urinary systems [1,2]. It has been implicated in lung [3,4], brain [5], and bladder cancer [6], increased blood pressure [7], altered neurological activity [8], increased thrombotic risk [9], increased stroke risk [10], increased risk of type 2 diabetes [11,12,13], and asthma [14]. Biodiesel exhaust shares many of the same physicochemical characteristics as diesel exhaust, such as oxides of nitrogen (NO_x_), carbon monoxide and dioxide, particulate matter consisting of mostly ultrafine particles [15,16,17], polycyclic aromatic hydrocarbons (PAHs), aldehydes, ketones, and heavy metals [18,19,20], and, thus, it is suspected that it will be associated with many of the same negative health impacts. Previous studies into engine and exhaust characteristics that compared diesel and biodiesel often show that biodiesel exhaust contains more NO_x_, PAHs, and ultrafine particles (<100 nm) but less overall particulate matter by weight [18,20,21,22,23,24] compared with mineral diesel exhaust. This is of concern as ultrafine particles, when compared to larger sizes, are more commonly linked to the negative health effects of air pollution [25,26]. Up to 90% of diesel exhaust particles by number consist of nucleation mode particles, newly formed from combustion and chemical reactions and are under 30 nm in size [15,16]. Thus, a further increase in the proportion of ultrafine particles in biodiesel exhaust is of great concern, in part due to increased surface-area-to-volume ratios allowing for more dangerous chemicals to be ab/adsorbed onto them for a given particle mass [22]. Despite this, biodiesel fuel usage is increasing worldwide [27].

Previous studies, both in vitro and in vivo, looking into the health effects of biodiesel exhaust typically use less than optimal exposure models [28,29]. Many previous studies have focused solely on the health effects of the exhaust particles by collecting them on a filter and adding a set concentration directly to a flask of cells/bacteria or instilling it into the nose/trachea of rats/mice [28,30,31,32,33]. While it allows an accurate dosage of particles to be given [33], in return, it both ignores the effects of the exhaust gases, which have their own set of negative health impacts [34], and also removes the ultrafine particles, arguably one of the most toxic components of diesel exhaust, which readily agglomerate on filters to form larger-sized particles [35]. This generates an artificial particle size spectrum, with previous studies showing that over a 16-fold increase in particle concentration is needed to generate the similar health impacts as if whole exhaust was used [36].

In addition to this, the majority of previous biodiesel exhaust toxicology studies have used bacterial AMES tests to study mutagenic effects, or have exposed immortalized cell lines [23,24,37,38] that are not always human or even derived from the respiratory system, the first exposed and likely most affected tissue [37,39]. Previous studies that expose animals most often use instillation to expose the mice/rats to the particulate matter in solution, with few studies performing inhalation exposures. These few studies are often divided into several different publications, likely due to the difficulty in conducting them, which artificially inflates the actual number of inhalation studies performed [40,41,42,43,44,45,46,47,48,49,50]. Of the few studies that do use inhalation exposures, only half expose mice/rats to pure biodiesel exhausts and the remainder use biodiesel blended with diesel (at ratios of <30% biodiesel in diesel fuel). With the biodiesel concentration being below half of the total fuel content, there is a chance for biodiesel-exhaust-exposure-induced health effects to be masked by those of diesel. That said, blended fuels are highly relevant to what is being used today with biodiesel already being blended up to 20% in some countries [51,52,53].

There is also a tendency in the literature to treat all biodiesels as the same, despite evidence that the feedstock used to make the biodiesel vastly affects the fuel and exhaust properties and, thus, the resulting health impacts of exhaust exposure [54]. Studies often use just one biodiesel type and make claims about biodiesel in general based on the results of that type [55]. Some studies do not even disclose the feedstock used to make their biodiesel [55,56]. Methodological differences inherent in different study designs—from the exact engine type, the use (or not) of exhaust after-treatment systems, differing exhaust concentrations, the use of whole exhaust compared to filter-extracted particles, and the wide range of health effects measured, including mutagenicity, cytotoxicity, and immunological effects [23,24,29,40,41,46,48,50,57]—make comparisons of fuel feedstocks between different studies difficult.

Thus, the aim of this study was to compare the respiratory health effects of exposure to one of two different types of biodiesel exhaust, using air and ultra-low sulfur mineral diesel (ULSD) as controls, to evaluate the impacts of biodiesel exhaust exposure and how these impacts can change between different feedstock types. Tallow and Canola were chosen for study, both because they are commonly used today [58,59] and previous research found them to be at extreme ends of the health effects in both a submerged and air–liquid-interface cell-culture exposure model [54,60]. Mice were exposed for two hours each day to one of these four options for eight consecutive days. The main hypotheses were that (i) exposure to Tallow biodiesel exhaust would result in more severe and a greater range of negative health effects than ULSD exhaust exposure and (ii) that exposure to Canola biodiesel exhaust would result in less health impacts.

## 2. Results

### 2.1. Exhaust Gas Characteristics

The mean and standard deviation for each fuel and gas type are shown in Table 1, with the exception of CO that showed only the highest reading at 10 min for each of the repeated exposures due to the cold-start effect on the performance of the catalytic converter. Trends over time can be found in the Supplementary Materials (Figure S1). All fuels displayed similar trends over time with NO_x_ (NO and NO_2_), CO_2_, and SO_2_ increasing rapidly in the first 30 min of the exposure, O_2_ decreasing rapidly in the first 20 min, and CO peaking in the first 10 min before decreasing rapidly to undetectable concentrations. Canola was found to be the most different of the tested fuels with significant changes in each of the measured combustion gases except for CO when compared to both Tallow biodiesel diesel exhaust (*p* < 0.05). In contrast, Tallow and ULSD exhaust were only different for O_2_, CO_2_, and SO_2_.

### 2.2. Exhaust Particle Characteristics

**Figure 1:**
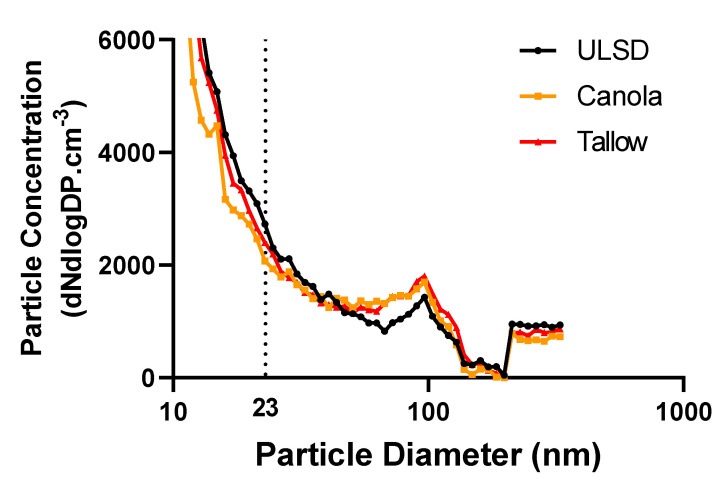
Particle size spectra representative of what each group of mice were exposed to for the three measured exhausts. Data were analyzed using total particle number concentration between the size of 10 and 340 nm for each fuel. Dotted line at 23 nm represents the approximate divide between solid and liquid particle formation [61]. No significant differences in particle number were found.

Particle size spectra were obtained for all exhausts between the sizes of 3–340 nm; however, no differences were observed for any of the fuels for total particle number concentrations (Figure 1). Particle mass concentrations (Table 1) were highest in ULSD; however, the concentrations in the Canola and Tallow biodiesels were 78% and 92% of those measurements, respectively, showing little differences between fuels.

### 2.3. Mouse Weights

**Figure 2:**
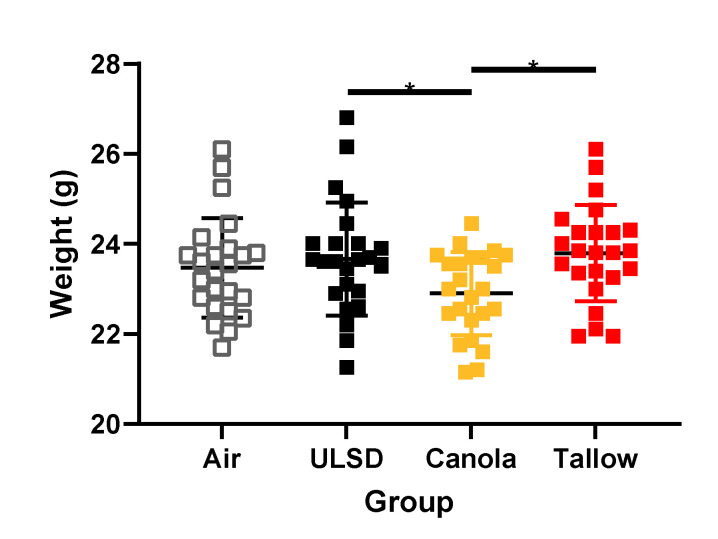
Mouse weights measured 24 h after the last exposure for each group (* indicates *p* < 0.05). *n* = 24 per treatment. Data were analyzed using general linear modeling methodologies.

Mice were weighed prior to any exposure, and again before lung function assessment, allowing for calculation of the weight changes between the exposure groups (Figure 2). On the day of study, Canola exhaust exposure weights were significantly less than those of ULSD and Tallow. No significant differences were found in % weight increase over the 8 days of exposure for any of the groups.

### 2.4. Lung Function at Functional Residual Capacity

Thoracic gas volume (TGV) and lung mechanics (airway resistance (R_aw_), tissue damping (G), tissue elastance (H), and η (hysteresivity)) at functional residual capacity (FRC) were measured (Table 2). TGV was significantly higher in Canola mice compared with all other groups. Due to this, lung function parameters at FRC were normalized to TGV to generate specific lung function measurements. Canola-exhaust-exposed mice had significantly higher sR_aw_ in comparison to ULSD and Tallow groups (*p* < 0.01). Specific G, H, and hysteresivity were significantly higher for Canola mice in comparison to all other groups (*p* < 0.01).

### 2.5. Volume Dependence of Lung Function

**Figure 3:**
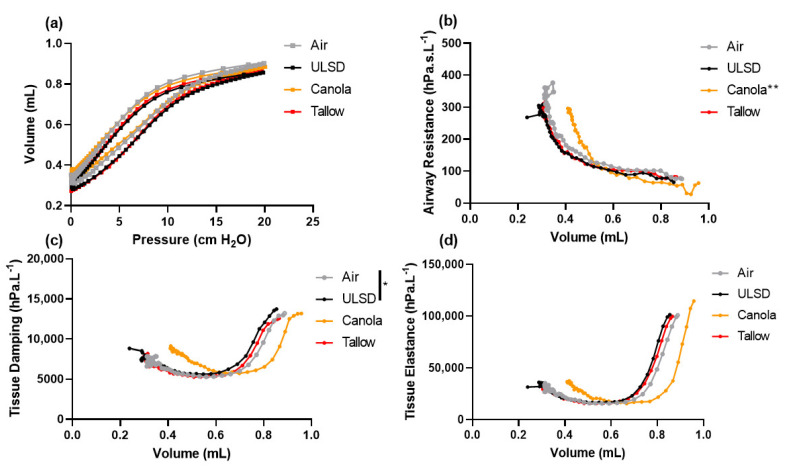
(**a**) Pressure–volume loops for mice exposed to Air (A), ULSD (U), Canola (C), or Tallow (T) biodiesel exhaust for 2 h/day for 8 days and volume dependence of lung function for (**b**) airway resistance, (**c**) tissue damping, and (**d**) tissue elastance for mice exposed to Air, ULSD, Canola, or Tallow biodiesel exhaust for 2h/day for 8 days. Data are group means. *n* = 23 for all groups except ULSD (*n* = 22) and Tallow (*n* = 21). Differences between groups were analyzed statistically at P_rs_ = 20 cm H_2_O or a lung volume of 0.7 mL, representing the highest volume for which data were available for each individual (* = *p* < 0.05, ** = *p* < 0.01, significant * values not located next to a line indicate that the group is significantly different to all other exposures). Data were analyzed using general linear modeling methodologies.

Respiratory pressure–volume curves and volume-dependent R_aw_, G, and H (Figure 3) were measured throughout a slow, induced inflation–deflation maneuver for each mouse. Specific compliance was significantly lower for Canola-biodiesel-exhaust-exposed mice compared to all other groups (*p* < 0.0001). At a volume of 0.7 mL (chosen as it is the largest lung volume with data for all mice), Canola-exposed mice were the most different to every other group, with significantly lower R_aw_ (*p* < 0.01), whereas ULSD-exposed mice showed significantly increased tissue damping compared to Air controls (*p* < 0.05).

### 2.6. Responsiveness to Methacholine

**Figure 4:**
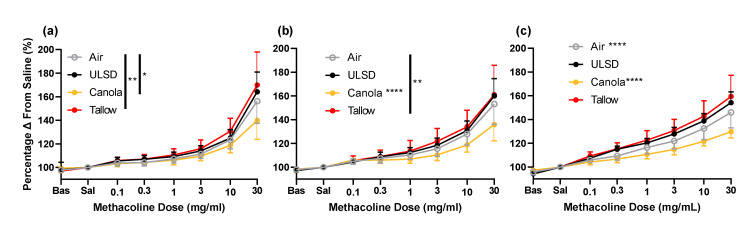
Responsiveness to methacholine for mice exposed to Air (A), ULSD (U), Canola (C), or Tallow (T) biodiesel exhaust for 2 h/day for 8 days. Data shown are (**a**) airway resistance, (**b**) tissue damping, and (**c**) tissue elastance for all exposure groups (*n* = 12, except Air and ULSD where *n* = 11). Bas = baseline (FRC), Sal = saline. All data are shown as % change from saline (* = *p* < 0.05, ** = *p* < 0.01, **** = *p* < 0.0001, significant * values not located to the right of a line indicate that the group is significantly different to all other exposures). Data were analyzed using general linear modeling methodologies.

**Figure 5:**
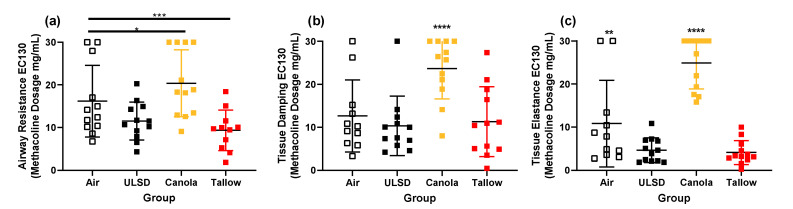
Evocative concentration showing methacholine dose needed to produce a 30% increase from saline in (**a**) airway resistance, (**b**) tissue damping, and (**c**) tissue elastance in mice exposed to Air, ULSD, Canola, or Tallow biodiesel exhaust for 2 hr/day for 8 days (*n* = 12, except Air and ULSD where *n* = 11) (* = *p* < 0.05, ** = *p* < 0.01, *** = *p* < 0.001, **** = *p* < 0.0001, significant * values not located next to a line indicate that the group is significantly different to all other exposures). Data were analyzed using general linear modeling methodologies.

R_aw_, G, and H were measured after exposure to increasing doses of methacholine (Figure 4). There were significant effects of treatment on the responsiveness to MCh with respect to airway resistance. Tallow mice were significantly more responsive than Air (*p* < 0.018) but Canola mice were significantly less responsive than all other groups (*p* < 0.001 in all cases). For G, Tallow mice were significantly more responsive than Air mice and Canola mice had significantly lower responses than all other groups (*p* < 0.01 in all cases). For H, all treatments were significantly different to each other with Tallow having the highest response and Canola the lowest (*p* < 0.0001 in all cases). This pattern was repeated in terms of sensitivity to MCh (evocative concentration needed to reach a 30% increase in R_aw_, G, and H from saline; Figure 5). The dose of MCh required to elicit a 30% increase in response was significantly lower in the Tallow mice for R_aw_ and H, significantly lower in the ULSD mice for H, and significantly higher in the Canola mice for R_aw_, G, and H when compared to Air mice (*p* < 0.05 in all cases).

### 2.7. Bronchoalveolar Lavage Cells, Mediators, Protein, and Phospholipid Concentrations

**Figure 6:**
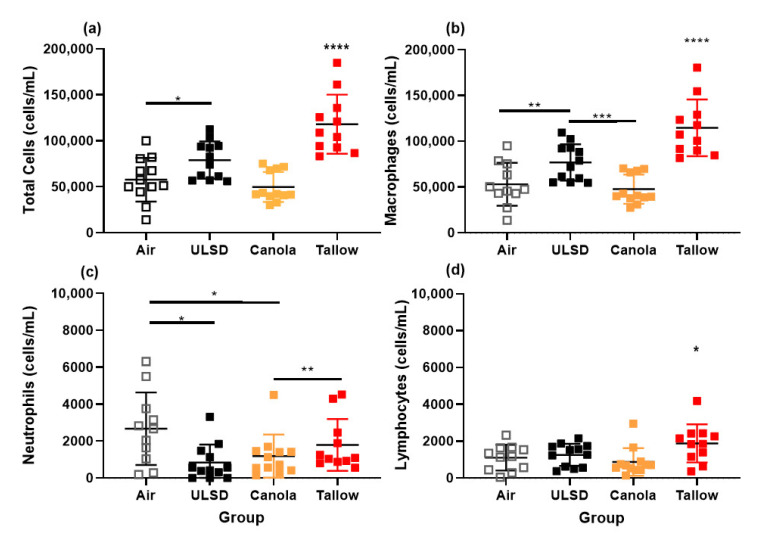
Cellular inflammation in bronchoalveolar lavage. Results are shown for (**a**) total cells, (**b**) macrophages, (**c**) neutrophils, and (**d**) lymphocytes (*n* = 12, except Tallow where *n* = 11) (* = *p* < 0.05, ** = *p* < 0.01, *** = *p* < 0.001, **** = *p* < 0.0001, significant * values not located next to a line indicate that the group is significantly different to all other exposures). Note different scales. Data were analyzed using general linear modeling methodologies.

**Figure 7:**
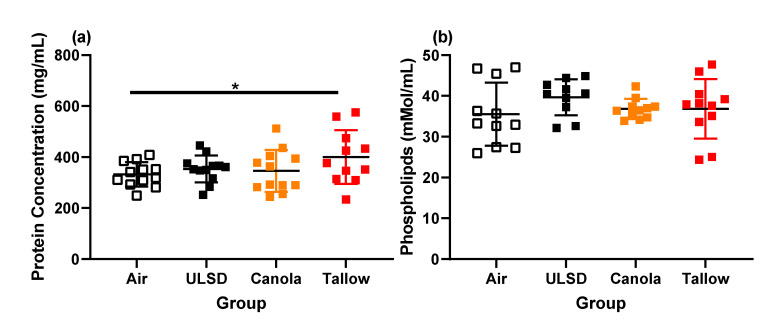
(**a**) Total protein concentration in the bronchoalveolar lavage and (**b**) total phospholipid concentration in the bronchoalveolar lavage for mice exposed to Air, ULSD, Canola, or Tallow biodiesel exhaust for 2 hr/day for 8 days. For protein, *n* = 12 except Tallow where *n* = 11. For phospholipid concentration, *n* = 11 except for ULSD where *n* = 10 (* = *p* < 0.05). Data were analyzed using general linear modeling methodologies. Data are individual mice with mean ± SD.

Total and differential cell counts were performed on bronchoalveolar lavage (Figure 6). Significantly more cells were found in the BAL of Tallow and ULSD mice compared to Air (*p* < 0.05). The Tallow group also had more cells than the Canola and ULSD mice (*p* < 0.05). This pattern was seen again in the macrophage cell counts, with the addition of ULSD having more macrophages in the BAL than Canola (*p* < 0.05). In contrast, the ULSD and Canola mice had significantly fewer neutrophils than the Air-exposed mice (*p* < 0.05). Tallow mice had more lymphocytes than the Air, Canola, and ULSD mice (*p* < 0.05). No other cell types were detected. In terms of BAL mediator concentrations (Table 3), the majority of significant differences was found between Tallow and Air, with 1 mediator being significantly increased and 3 mediators significantly decreased for Tallow-biodiesel-exhaust-exposed mice (*p* < 0.05). Total protein and phospholipid concentrations within the BAL were also measured (Figure 7). There were few effects of exposure on either of these parameters; however, Tallow mice had a significantly increased protein concentration in comparison with Air mice (*p* < 0.05).

### 2.8. Multivariate Analysis

**Figure 8:**
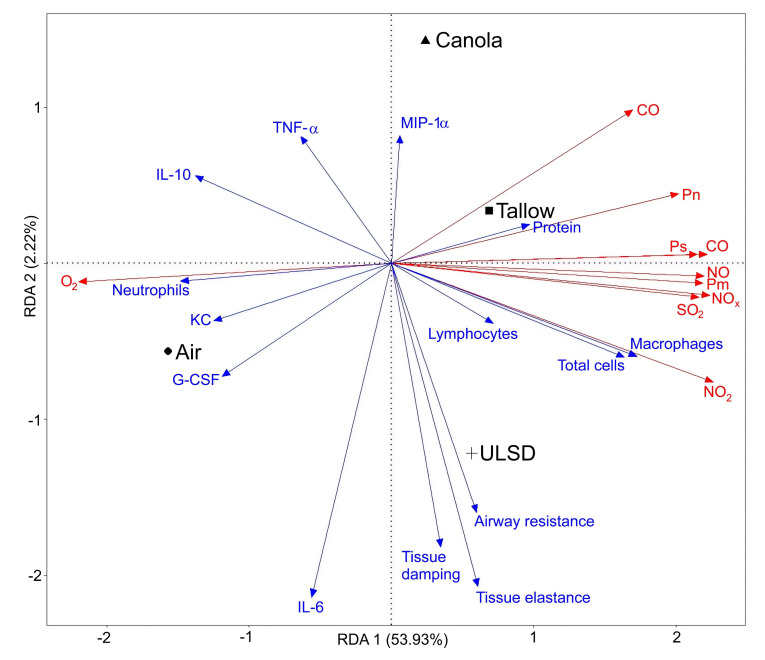
Redundancy analysis model plot showing correlations between exhaust components and important toxicological endpoints. The percentages for each axis are the proportion of variability explained by that axis for the entire dataset; thus, for this model, movement along the *x*-axis has a greater importance than movement along the *y*-axis. Exhaust variables are in red, toxicological outcomes are in blue. Arrows that point in the same or opposite directions are highly correlated (positively or negatively, respectively), whereas arrows that point perpendicularly indicate little or no correlation between parameters. Note: Pn = particle number, Ps = particle size, Pm = particle mass.

Fourteen key outcomes obtained from the responsiveness to methacholine analyses (maximum response in airway resistance, tissue damping, and tissue elastance) and bronchoalveolar lavage (total cellular inflammation, numbers of macrophages, neutrophils and lymphocytes, levels of protein, G-CSF, IL-6, IL-10, KC, MIP-1α, and TNF-α) were fitted to the ten exhaust variables (levels of gases O_2_, CO, CO_2_, NO, NO_2_, NO_x_, and SO_2_, in addition to particle size, number, and mass) using a redundancy analysis (RDA) (Figure 8) in order to assess how well the exhaust components explained the resulting biological results. Due to inherent limitations of RDA, only data with complete datasets for all mice in all exhaust-exposed groups were able to be included in analyses. To not overfit the model by overloading it with all cytokine data available, six cytokines were chosen from the 21 analyzed mediator levels. These were chosen based on either their importance in previous exhaust toxicology studies [1,22,28,54] (IL-6, IL-10, TNF- α) or for observed significant differences between exhaust groups and the air control (G-CSF, KC, MIP-1 α). The fitted RDA model showed that over half (56.98%) of the biological variability observed in responsiveness to MCh and BAL outcomes was explained by the exhaust parameters. This type of analysis is a powerful tool in supporting the validity of our findings as it shows strong correlations between related parameters. For example, neutrophilic inflammation is strongly positively correlated with levels of KC (a neutrophilic chemoattractant mediator). Similarly, the responsiveness to MCh outcomes (R_aw_, G, and H) are all positively correlated with each other, as are the majority of exhaust gases. The oxygen concentration in exhaust is negatively correlated with all other exhaust components, which are strongly correlated with each other (as indicated by the red arrows in Figure 8). This is unsurprising due to fuel combustion requiring oxygen for the reaction. Additionally, neutrophilic inflammation is correlated with neither the total number of inflammatory cells in the bronchoalveolar lavage nor the numbers of other cell types (macrophages and lymphocytes); instead, it is highly negatively correlated with particle number and CO_2_. The total cell count is highly correlated with NO_2_ concentrations. Perhaps most importantly, many of the mediator and methacholine response measurements are not correlated with any one exhaust component, suggesting the more complex relationship between exposure and toxicological result that is not currently explainable by the inputted data.

### 2.9. Airway Morphometry

Size-corrected total airway wall, airway smooth muscle mass, and airway epithelial thickness were measured (Table 4, representative images in Figure S2). Chord length, collagen, and total tissue % were also measured (Table 4). There was no effect of treatment on any airway morphometry parameter (*p* > 0.05 in all cases). Chord length was significantly higher in Tallow mice compared to Air mice, and Tallow mice had significantly more collagen than Canola and ULSD mice did, suggesting that exposure to Tallow biodiesel exhaust was causing measurable damage to the airways (*p* < 0.05).

## 3. Discussion

The results of this study show that exposure to diluted diesel or biodiesel exhaust causes a range of negative health impacts in a murine exposure model. These include impacts on lung function, cellular inflammation, small changes to lung structure, and large impacts to the immune response. Of the two biodiesels tested, Tallow biodiesel exhaust exposure was associated with the widest range of negative health effects with a greater increase in responsiveness to methacholine, a greater than two-fold increase in inflammatory cell numbers in the lungs, a wider disruption in the local mediator release of the lungs, increased protein concentrations in the BAL and a small impact on lung structure with significantly increased chord length. In contrast, Canola biodiesel exhaust exposure only led to negative impacts on lung function at FRC, specific compliance, some decreases in mediator release, and decreased neutrophilic (but not total) inflammation. Mice exposed to Canola biodiesel exhaust were less responsive to MCh than Air-exposed controls, an interesting finding for which we do not have an explanation. The impacts of exposure to ULSD exhaust were generally between those of Canola and Tallow, with increased tissue damping in volume-dependent lung mechanics, several increases in methacholine responses, some decreases in mediator release, and increased immune cell numbers in the lungs of exposed mice.

A concerning implication for this study is that negative health impacts (with implications for wide-reaching consequences) were identified, yet the exhaust used mostly met Safe Work Australia standards [62]. These standards are equivalent to the standards used in Europe and USA [63,64]. The Safe Work Australia standards for various exhaust components are time-weighted 8 h averages of 30 ppm of CO, 5000 ppm of CO_2_ (peak concentration not exceeding 30,000 ppm), 25 ppm of NO, 3 ppm of NO_2_ (with peak concentrations not exceeding 5 ppm), and 2 ppm of SO_2_ (peak concentration not exceeding 5 ppm). Oxygen levels below 19.5% are considered “unsafe” [65]. Table 1 shows that almost all exhaust gases in this study (with the exception of a slightly too high NO_2_ and a slightly too low oxygen concentration) met these standards. The European Union has set a recent particulate matter occupational exposure limit of 50 ug/m^3^ of elemental carbon [66,67], whereas in America, the limit for a non-coal mining setting is set at 160 µg/m^3^ of total carbon [68], and in Australia, it is recommended that diesel exhaust not exceed 100 µg/m^3^ of elemental carbon [69]. In this study, particle mass concentrations were between 42.6 and 54.4 µg/m^3^, again showing that common exposure standards were not exceeded.

In the current study, biodiesel exhaust did not contain higher levels of NO_x_ or lower levels of PM compared with ULSD exhaust, as is commonly reported in the literature [18,19,20,22,70]. That said, other studies that measured NO_x_ and PM have also reported either no differences between biodiesel and mineral diesel exhausts, or a decrease in the biodiesel exhaust [19,47,55]. Our previous studies have also found a wide variation in NO_x_ and PM concentrations in biodiesel exhaust compared to ULSD, with differences dependent on the feedstock type used to make the biodiesel [22,54,71]. This suggests that differences in NO_x_ and PM concentrations between diesel and biodiesel are subtle enough that the dilutions used in toxicology studies to make concentrations “real-world”-relevant can mask the changes [18,20,54] and/or that the differences are related to feedstock type. The overarching idea in the literature that biodiesel exhaust overall contains more NO_x_ and less PM may be feedstock-specific and should, thus, be viewed critically [54]. This idea is further supported by the findings that Tallow biodiesel exhaust is no different to ULSD in terms of PM and NO_x_, but that Canola biodiesel exhaust contained significantly less NO_x_. Another potential explanation is that many previously published exhaust-only comparisons have been conducted using old technology engines not equipped with a diesel particulate filter and/or diesel oxidation catalyst [18,20,23] and, thus, increased NO_x_ and decreased PM in biodiesel exhaust may only be applicable to older technology engines [19,47,55]. Regardless, the multivariate RDA results suggest a correlation between exhaust gas components and several toxicological responses, showing that it is important that any future experiments analyzing the toxicity of diesel or biodiesel exhaust should use whole exhaust exposure methods instead of focusing solely on the health effects of exhaust particles alone, as is too often conducted in the previous literature [1,28]. For example, we identified a strong correlation between NO_2_ and inflammation in the form of total cells and the number of macrophages present in the BAL (Figure 8). This correlation is not surprising based on the known inflammatory effects of NO_2_ [72].

A key finding of this study is that the Tallow-biodiesel-exhaust-exposed mice were hyperresponsive to MCh with respect to airway resistance, tissue damping, and tissue elastance. The mice in this study were exposed for only two hours per day for 8 days, to exhaust that largely met Safe Work Australia Standards, and yet responsiveness to methacholine increased significantly. The response measured is smaller compared to similar exposure studies in smoking, asthmatic, and respiratory viral infection mouse models [73,74,75]; however, comparisons between models that employ a variety of environmental exposures are difficult. Previous studies testing the response to methacholine in mice after intranasal instillation of black carbon or diesel exhaust found a greater hyperresponsiveness than was measured in our study, although differences in diesel exhaust exposure protocols and methacholine dosages make direct comparisons difficult [76,77]. Studies that co-exposed house dust mites and diesel exhaust also found increased responsiveness to methacholine, although only in the co-exposed group and not in the diesel-exhaust-alone-exposed group [78,79]. Studies testing the response to methacholine after diesel exhaust exposure in asthma and atopy also found increased hyperresponsiveness, although, once again, these studies cannot be directly compared, due to differences in subject type and measurements [80,81]. Our finding of increased airway hyperresponsiveness has concerning implications for those with asthma and allergies who are currently exposed for prolonged periods of time to diesel exhaust, as the increased responsiveness to methacholine suggests that a swap to using some biodiesel feedstocks for fuel may elicit worse responses. Additionally, diesel exhaust can act as a sensitizer to aeroallergens and our data suggest that Tallow-derived biodiesel may further enhance that effect [82]. The results of the RDA (Figure 8) suggest that the relationship between exhaust exposure and methacholine response is not straightforward, nor is it correlated with any one particular exhaust component, unlike the measurement of protein concentration present in the BAL, which is highly correlated with the number of particles present in the exhaust.

The finding that mice exposed to Canola biodiesel exhaust for 2 h per day for 8 days were less responsive to methacholine than Air controls was unexpected. Despite being the least toxic in terms of methacholine response and pulmonary cellular inflammation, the Canola biodiesel exposure group displayed both positive and negative health impacts. While the lower 8-day methacholine responsiveness compared to Air, increased thoracic gas volume measurements (despite the Canola mice being significantly smaller than the other groups) and decreased airway resistance in the volume-dependent measurements could be interpreted as positive findings (i.e., “improvements” compared with Air controls), when combined with the negative indications of increased specific R_aw_, G, and H at FRC, it instead suggests that the complete picture is much more complicated. Diesel (and, thus, likely biodiesel) exhaust is a highly complex mixture made up of thousands of different chemicals [18,37,57,83,84,85,86] and it is possible (and indeed likely, from the results of this study) that exposure to such a mixture could lead to both “positive” and “negative” health impacts as seen for Canola biodiesel exhaust. Further experiments are needed to explore what makes the Canola biodiesel exhaust exposure group so unique. Such research could identify what is changing in the lungs of exposed mice, and also what component(s) of the Canola biodiesel exhaust are associated with the changes. We attempted to address this by examining surfactant levels in the lungs via measurement of BAL choline containing a phospholipid concentration. The surfactant is comprised of approximately 70% of phosphatidylcholine, which, in turn, makes up approximately 80% of phosphatidylcholine in the lungs [87,88]. It is both produced naturally and also used medicinally to improve breathing in preterm children and other children at risk of respiratory failure, as it acts to decrease surface tension at the air–liquid interface of the lung alveoli [88]. However, no difference in phospholipid concentrations was found. Thus, reasons for why the Canola mice responded as they did are difficult to elucidate and warrants further investigation.

Another key finding of this study was the increased cell numbers in the BAL of Tallow biodiesel and ULSD-exhaust-exposed mice. This increase mostly consisted of an increase in macrophages, and an increase in lymphocytes in the Tallow-exposed group. A decrease in neutrophils was also observed in the Air group compared to both the Canola and ULSD groups, a finding that is further supported by the RDA showing negative correlations between total cell count and neutrophil count. This suggests that some immune dysregulation might be occurring in mice sub-chronically exposed to exhaust, a finding that is supported by the local (BAL) mediator response that shows significant decreases in the Tallow-exposed mice compared to their respective Air controls. Due to kinetics of immune mediator release after exhaust exposure, wherein the greatest immune responses in a previous study were found 3–6 h after exposure with decreases back to baseline levels observed by 24 h [89], a decrease after a single day of exposure could be explained as immune mediators being “used up”. However, with the depletion effect ongoing after 8 days of exhaust exposure, combined with the decrease in neutrophil numbers in all groups, even if this decrease was only statistically significant for Canola and ULSD, this instead suggests an inability for the mouse immune system to cope with ongoing exhaust exposure, which could have serious consequences for cancer and infection [90,91,92,93]. These findings have been mirrored in a diesel exhaust human exposure study of occupationally exposed workers [90], which found workers exposed to high amounts of exhaust for prolonged periods showed immune dysregulation and decreases in serum inflammatory mediators, such as IL-8 and MIP-1β. In addition, previous studies co-exposing mice to both a respiratory infection and diesel exhaust found that exposure increased infection susceptibility [93,94]. Studies have also been able to induce allergic airways disease using diesel exhaust [82] and human exposure studies on populations with allergic rhinitis found that diesel exhaust exacerbated allergic inflammation, likely by dysregulating the immune systems’ ability to remove eosinophils [95].

There were also minor changes in the lung structure of Tallow-biodiesel-exhaust-exposed mice in terms of a small but statistically significant increase in chord length. Chord length, also known as mean linear intercept, is a measure of the mean space between airway structures [96,97], and increased chord length has been linked to airway damage and disease such as emphysema [98], although it is not a direct measurement of airway size [96]. The Tallow-exposed mice also had increased protein content in the BAL, which is a marker of increased lung permeability and epithelial damage [76,99] and further supports our previous finding of increased epithelial cell damage and increased permeability in air–liquid-interface cell cultures [60]. Increased epithelial damage and increased chord length would indicate damage to the airways after Tallow biodiesel exhaust exposure [97,98], which is concerning after such a relatively short-duration exposure period. There were no other indications of changes to airway morphometry; however, very mild exposures were used in comparison to some previous studies [41,42,100].

## 4. Materials and Methods

### 4.1. Animals

**Figure 9:**
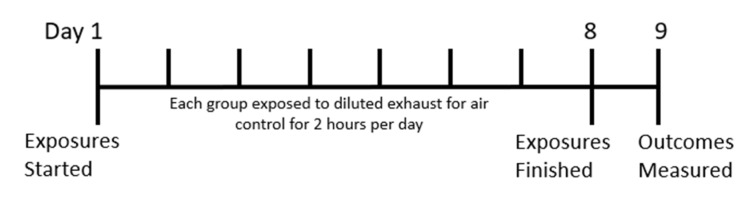
Exposure timeline. Each group was exposed for two hours once a day for 8 days to the same diluted exhausts or Air as a control. Measurements were taken 24 h after the last exposure.

Ninety-six seven-week-old male BALB/c mice were purchased from the Animal Resources Center (Murdoch, WA, Australia) and housed in individually vented cages (IVC Allentown XJ model, ECO FLO Air handling unit set at 22–23 °C with 30–31% humidity, 50 air changes per hour). They were left to acclimatize for one week before being weighed and randomly assigned into one of 4 different groups (*n* = 24 per group). These groups were exposed for 2 h per day for 8 days to Air or the diluted exhaust of an engine running on ULSD, Canola, or Tallow biodiesel (Figure 9). Twenty-four hours after the last exposure, mice were weighed and prepared for end exposure outcomes as previously described [101].

### 4.2. Engine Configuration and Fuel Information

Exhaust was generated by a single-cylinder, 435 cc design Yanmar L100V engine (Yanmar, Italy) coupled with an electric fuel pump with multistage filtration and a dynamometer and fitted with Euro V/VI after-treatment technology consisting of a diesel particulate filter and oxidation catalyst (Daimler, Germany) [71]. All exposures were run from cold start with a constant load of 40% and a speed of 2000 rpm. Air exposures were conducted simultaneously alongside exhaust exposures. The diesel fuel used as the control was obtained from a local supplier (SHELL, Australia, <10 ppm sulfur). Both Canola and Tallow biodiesel were created following an established sodium methoxide transesterification process [102] using high-quality oils obtained from local suppliers (Campbells Wholesale Reseller, WA, Aus and Range Products, WA, Aus). Detailed FAME profiles have been previously published [54,103]. The diesel exhaust exposure consumed less fuel due than was required for both biodiesel exposures, likely due to the differences in fuel efficiency [18].

### 4.3. Exposure Protocol

**Figure 10:**
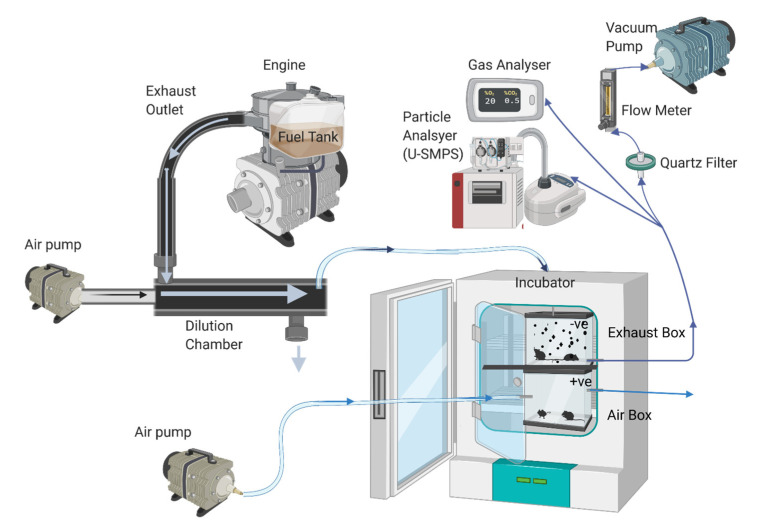
Diagram of exposure setup. As a vacuum is used to transport exhaust into the exhaust chamber and a pump is used to transport air into the Air chamber, a slight variation in pressure gradient was created to ensure there was no chance for cross-exposure of groups. Created with Biorender.com.

The exposure methodology is based on a combination of previously published protocols [41,103]. To make exposures more realistic to occupational settings, mice were exposed for short time periods to exhaust diluted approximately 1/10 with air with cold-start emissions included as part of the exposure. The exhaust was diluted inside a mixing chamber attached to the exhaust piping and pumped through an isokinetic sampling point at a rate of 5 L/min into a sealed incubator (Model 1535, Sheldon Manufacturing, OR, USA) maintained at 28 °C containing a 27 L exposure chamber with the mice inside divided into individual cubicles. The sealed incubator was used to dampen the sound of the engine and keep chamber temperatures constant. During exposures, exhaust was gently vacuumed out of the exposure chamber for physicochemical analysis of gas and particle properties (Figure 10). Simultaneously to the exhaust exposure, a second 4 L exposure chamber was also placed inside the incubator and attached to piping that allowed air to be pumped inside for the Air exposure controls. The difference of pumping air into and vacuuming exhaust out of the different chamber boxes created a pressure gradient that made certain of no chance for cross-exposure contamination, in case of any leakages within the sealed exposure chambers. Fewer Air mice were exposed at any one time (i) because of the smaller control exposure chamber and (ii) to ensure that there were control animals on each data acquisition day. All exposure chambers were thoroughly washed and dried between exposures.

### 4.4. Gas and Particle Analysis

Exhaust exiting the exposure chamber was analyzed at the beginning of every exposure and then every 10 min until the end of exposure for concentrations of combustion gas products including O_2_, NO_x_ (NO and NO_2_), CO, CO_2_, and SO_2_ using a combustion gas analyzer (TESTO 350, Testo, Lenzkirch, Germany). Similarly, exhaust was analyzed every 10 min for particle concentrations between the sizes of 3 nm and 340 nm using a Universal Scanning Mobility Particle Sizer (U-SMPS 1700 Palas, Karlsruhe, Germany). Particles less than 10 nm in size were excluded from further calculations due to the high variability of measurements at that size range. Median particle size count was calculated using the mean number of particles. Particle mass was calculated from particle spectra, assuming sphericity and using the 40% load diesel exhaust particle density as previously described [104]. Particle number was further separated into two fractions: nucleation mode particles below 23 nm in size and solid particles above 23 nm [61]. Due to the high dilution and aftertreatment devices used in this experiment, there was not enough particulate matter collected on quartz filters to perform detailed chemical analysis on polycyclic aromantic hydrocarbon, aldehyde, or heavy metal concentrations present in the exhaust [60].

### 4.5. Lung Function Measurements

Measurements of thoracic gas volume (TGV) and lung mechanics were conducted as previously described [73,105,106]. In brief, mice were anesthetized via intraperitoneal injection of a solution containing ketamine (40 mg/mL; Troy Laboratories, New South Wales, Australia) and xylazine (2 mg/mL; Troy Laboratories, New South Wales, Australia) at a dose of 0.1 mL/10 g body weight, tracheostomized with a 10 mm long cannula with an internal diameter of 0.86 mm, and attached to a mechanical ventilator (HSE Harvard Minivent; Hugo Sachs Harvard Elektronik, March-Hugstetten, Germany). They were ventilated at a rate of 400 breaths/min with a tidal volume of 8 mL/kg and 2 cmH_2_O of positive-end expiratory pressure, which is sufficient to allow measurement of lung function parameters without either induction of paralysis or autonomous breathing. Plethysmography was used to measure TGV. At end expiration, the trachea was occluded and the intercostal muscles electrically stimulated (six 2 to 3 ms, 20 V pulses, model S44 electrical stimulator; Grass Instruments, Quincy, MA, USA) to induce inspiration with tracheal pressure and plethysmograph box pressure measured throughout. TGV was then calculated using Boyle’s law, after correction for thermal properties and impedance of the plethysmograph [106]. Respiratory system impedance (Z_rs_) was measured using a wave-tube system adapted for use in small animals [107,108] and a modification of the forced oscillation technique [108]. The constant-phase model was fit to Z_rs_ to generate the parameters of airway resistance (R_aw_), tissue damping (G), and tissue elastance (H). Z_rs_ was measured at functional respiratory capacity and also during a slow inflation–deflation maneuver from 0 to 20 cmH_2_O transrespiratory pressure (P_rs_), allowing for construction of absolute pressure–volume curves and assessment of the volume dependence of lung mechanics. Specific lung compliance was then calculated between P_rs_ = 8 cm/H_2_O and 3 cm/H_2_O on the deflationary arm [109].

### 4.6. Methacholine Challenge

After measurement of TGV and lung mechanics, a randomized selection of half the mice from each group (*n* = 12) were transferred to a small animal ventilator (Legacy flexiVent; SCIREQ, Montreal, QC, Canada) for assessment of responsiveness to methacholine (MCh; acetyl β-methacholine chloride; Sigma-Aldrich, St. Louis, MO, USA) as previously described [110]. Briefly, 5x forced oscillation technique (FOT) measurements were taken at FRC/baseline (1 per minute), then after a 10 s saline aerosol and again after increasing doses of MCh from 0.1 to 30 mg/mL. Peak responses to MCh at each dose were used to construct dose–response curves.

### 4.7. Bronchoalveolar Lavage (BAL) Collection and Cell Measurement

At the end of the methacholine challenge, BAL fluid was collected by washing 0.5 mL of chilled saline in and out of the lungs three times via the tracheal cannula (*n* = 12 per group). Lavage samples were processed as previously described for total cell counts [111] and differential cell counts were obtained using DiffQuik (Thermofisher Scientific, Waltham, MA, USA) staining as per the manufacturer’s protocol. In short, samples were centrifuged at 400× *g* for 4 min to pellet the cells and the supernatant was removed and stored at −80 °C for future mediator, protein, and phospholipid analysis. A total cell count was determined from the cell pellet by staining an aliquot of cells with trypan blue and counting cells with a hemocytometer. Remaining cells were cytospun, stained with DiffQuik, and scanned using a Panoramic MIDI^®^ scanner (3DHISTECH Ltd., Budapest, Hungary) prior to visualization using ImageJ [112] to determine the proportion of cell types within a randomized count of 300 cells.

### 4.8. Lung Fixation, Airway Morphometry, and Histology

Non-methacholine-challenged mice had lungs inflation fixed at P_rs_ = 10 cmH_2_O using 10% formalin [96] prior to removal *en bloc* for airway morphometry analysis (*n* = 12 for all groups). The left lung was embedded in paraffin, and 5 μm thick sections were taken from the proximal region, where the primary bronchi were first fully enclosed by tissue. Three sections from each mouse were stained using Masson’s trichome and the most intact sections were imaged using a Panoramic MIDI^®^ scanner (3DHISTECH Ltd., Budapest, Hungary). Semiautomated assessment of chord length was performed [97,113] and collagen content was quantified as a percentage of total tissue in the cross-sectional area using ImageJ [112]. The cross-sectional area of the outside bronchi wall, airway smooth muscle, the gap between smooth muscle and the epithelium, and the airway epithelium were measured. The square root of all areas was normalized to the internal perimeter of the basement membrane to correct for differences in airway size [114].

### 4.9. Mediator, Phospholipid, and Protein Analysis

BAL was analyzed for mediators as per kit protocol using Bio-Rad Mouse Cytokine 23plx kits (Bio-Rad) and accompanying software (Bio-Plex Manager, v6.1.1, Bio-Rad, Tokyo, Japan). The protein concentration of BAL was used as an indirect measurement of airway epithelial damage and was assessed as per kit protocol using a Pierce™ BCA protein assay kit (Thermofisher Scientific). Phospholipid (choline containing) concentration within the BAL was analyzed to assess surfactant concentrations as per kit protocol using a Colorimetric Phospholipid Assay Kit (Abcam). Serum immunoglobulin concentration was analyzed as per kit protocol using Mouse Immunoglobulin Isotyping Magnetic Bead Panel (Milliplex, MERCK).

### 4.10. Statistical Analysis

Data are presented as mean ± standard deviation. This study was performed as part of a larger study with both 1-day and 8-day exposure groups. As the study was initially designed to have exposure groups for both days, statistical analyses were performed on the whole dataset (including both timepoints). Due to the complexity of the data, this larger study has now been separated into two. The 8 days of exposure results are reported in this paper. All statistical analyses were performed using R statistical software (v3.4.3) [115] loaded with the packages “lme4”, “mgcv”, and “Vegan” [116]. *p*-values less than 0.05 were considered significant. All statistical analyses excluding gas concentration data were completed using multivariate general linear modeling methodologies with the families “Gamma(inverse/log)” and “gaussian(identity/log)” as best fits to the data, applying a backwards elimination approach to remove insignificant predictive variables. For combustion gas analysis, a separate General Additive Model (GAM) file was fitted to each gas measurement with concentration as the response variable and time as the predictor, thus allowing for non-parametric fits as caused by cold-start effects. Redundancy analysis (RDA) [116,117] was performed using the package “Vegan” and a previously published protocol, inputting data obtained only from the methacholine challenge and the bronchoalveolar lavage due to the limitations of the model’s ability to deal handle missing data values, meaning that only whole datasets could be appropriately analyzed. This excluded both the histology data and lung function measurements at FRC due to different sets of mice being used for different measurements, and the mice used for airway morphometry measurements were not the same group as those used for the remainder of the results. To not overfit the model with the highly correlated cytokine data, 6 cytokines were chosen for analysis: IL-6, IL-10, G-CSF, KC, MIP-1α, and TNF-α chosen for either their importance to the innate immune response or for the observed significant differences compared to air-exposed controls [88,89,90,91,92,93,94]. Before completing the RDA, data were standardized using the command “decostand” to help account for different units of measurement used for different values [116,117]. The RDA was then performed as recommended by the “Vegan” package guidelines.

## 5. Conclusions

Exposure to diesel and/or biodiesel exhaust impacted lung function measured at FRC, volume-dependent lung function, methacholine responsiveness, inflammation, and airway morphometry in a mouse model. In line with our previous research [54,60]. Tallow biodiesel exhaust exposure resulted in the widest range of negative health impacts, followed by ULSD exhaust with Canola biodiesel exhaust causing the most limited impacts and arguably even having a positive effect on methacholine response. More research is needed to parse out reasons for this.

## Figures and Tables

**Table 1:** Mean (standard deviation) combustion gas concentrations and mean particle characteristics representative of what each group of mice were exposed to for the three measured exhausts. Measurements are shown as the mean concentrations for all of the exposures, with the exception of CO, which is shown as the mean peak measurement. Data in square brackets are ratios in comparison to ULSD, particle data in parentheses are percentages of the total within the fuel. Data were analyzed using general additive modeling methodologies.

	ULSD	Canola	Tallow
O_2_ (%)	19.45 (0.53)	19.58 (0.43) * ####	19.20 (0.49) **** ####
CO (ppm)	0.99 (0.76)	1.87 (0.90)	2.06 (1.15)
CO_2_ (%)	0.95 (0.35)	0.84 (0.27) ** ####	1.11 (0.32) **** ####
NO_x_ (ppm)	33.30 (14.52)	24.73 (9.35) **** ####	32.37 (13.37) ####
NO (ppm)	28.23 (11.48)	22.71 (8.42) **** ###	27.11 (9.95) ###
NO_2_ (ppm)	5.07 (3.27)	2.17 (1.47) **** ####	5.47 (3.87) ####
SO_2_ (ppm)	1.64 (0.73)	1.21 (0.53) **** #	1.38 (0.72) ** #
Particle Mass Concentration (µg/m^3^)	54.42	42.58 [0.78]	50.17 [0.92]
Median Particle Size (nm)	18	20	20
Total Particle Number (particles/cm^3^)	101,788	89,086 [0.88]	98,418 [0.97]
Particle Number >23 nm (particles/cm^3^)	39,035 (38.55%)	39,141 (43.94%)	41,191 (41.85%)
Particle Number <23 nm (particles/cm^3^)	62,753 (61.65%)	49,945 (56.06%)	57,228 (58.15%)

* = Different to ULSD (* = *p* < 0.05, ** = *p* < 0.01, **** = *p* < 0.0001). # = Different to the other biodiesel (# = *p* < 0.05, ### = *p* < 0.001, #### = *p* < 0.0001).

**Table 2:** Mean (standard deviation) thoracic gas volume (TGV) and specific lung function at FRC for mice exposed to Air, ULSD, Canola, or Tallow biodiesel exhaust for 8 days as measured by plethysmography and the forced oscillation technique (*n* = 22–24 per group). As significant differences in TGV were measured between groups, FRC lung function measurements have been normalized to TGV. Data were analyzed using general linear modeling methodologies.

FRC Lung Function Measure	Exposure			
	Air	ULSD	Canola	Tallow
TGV (mL)	0.291 (0.043)	0.279 (0.042)	0.328 (0.049) **###a	0.283 (0.035) a
sR_aw_, hPa·s^−1^	108.3 (23.80)	102.6 (20.82)	120.0 (21.08) ##a	103.6 (22.32) a
sG, hPa	2267 (421)	2281 (255)	2865 (398) ****#### a	2160 (322) a
sH, hPa	9446 (1734)	9656 (1248)	11,362 (1716) ***##a	9217 (1835) a
η	0.243 (0.020)	0.237 (0.015)	0.253 (0.024) **####a	0.236 (0.027)

TGV = thoracic gas volume; sR_aw_ = specific airway resistance; sG = specific tissue damping; sH = specific tissue elastance; η = hysteresivity. * = Different to Air controls (** = *p* < 0.01, *** = *p* < 0.001, **** = *p* < 0.0001); # = Different to ULSD (## = *p* < 0.01, ### = *p* < 0.001, #### = *p* < 0.0001); a = Different to other biodiesels (*p* < 0.05).

**Table 3:** Mean (standard deviation) mediator levels in bronchoalveolar lavage fluid for mice exposed to Air, ULSD, Canola, or Tallow biodiesel exhaust for 2 hr/day for 8 days (*n* = 12). Data were analyzed using general linear modeling methodologies.

BAL Mediator	8 Days Exposure			
	Air	ULSD	Canola	Tallow
IL-1α (pg/mL)	2.407 (0.806)	2.278 (0.967)	2.805 (1.097) a	1.976 (0.767) a
IL-2 (pg/mL)	2.835 (1.129)	2.642 (1.242)	3.643 (1.458) #a	2.176 (0.771) a
IL-4 (pg/mL)	0.338 (0.120)	0.305 (0.130)	0.357 (0.176) a	0.220 (0.091) *a
IL-5 (pg/mL)	0.900 (0.401)	0.697 (0.366)	0.990 (0.417)	0.795 (0.615)
IL-6 (pg/mL)	0.937 (0.744)	1.052 (0.743)	0.207 (0.070)	0.811 (0.722)
IL-9 (pg/mL)	3.364 (2.069)	3.351 (1.958)	3.795 (2.239)	2.749 (1.380)
IL-10 (pg/mL)	4.540 (1.297)	3.036 (1.365) *	4.160 (1.940) a	2.890 (1.461) **a
IL-12(p40) (pg/mL)	39.924 (16.53)	37.253 (7.705)	36.263 (8.970)	43.284 (17.725)
IL-12(p70) (pg/mL)	11.478 (2.881)	8.457 (4.369)	10.218 (4.897)	7.948 (5.217)
IL-13 (pg/mL)	17.758 (5.711)	15.983 (6.676)	21.243 (10.470)	15.875 (6.435)
IL-17 (pg/mL)	1.760 (0.343)	1.464 (0.487)	2.005 (0.864) #a	1.198 (0.470) *
Eotaxin (pg/mL)	5.084 (1.469)	4.215 (1.585)	5.359 (2.630)	4.625 (1.226)
G-CSF (pg/mL)	4.447 (2.192)	3.342 (1.101)	3.024 (0.009) *a	4.707 (3.623) a
GM-CSF (pg/mL)	4.494 (2.114)	3.903 (2.182)	4.725 (2.281)	3.495 (1.313)
IFN-γ (pg/mL)	3.201 (1.257)	2.711 (1.083)	3.152 (1.112)	2.495 (1.140)
KC (pg/mL)	39.244 (12.641)	30.240 (7.664) *	30.379 (2.714) *a	41.86 (21.676) #a
MCP-1 (pg/mL)	13.565 (8.868)	13.646 (7.047)	13.594 (8.997)	14.862 (12.742)
MIP-1α (pg/mL)	2.258 (0.584)	1.545 (0.457)	2.299 (1.297) a	3.945 (3.918) *#a
MIP-1β (pg/mL)	11.348 (4.568)	8.628 (6.085)	9.672 (6.022)	8.876 (7.383)
RANTES (pg/mL)	8.276 (2.247)	7.515 (3.282)	9.357 (3.376) a	6.410 (2.617) a
TNF-α (pg/mL)	8.913 (2.940)	7.345 (2.861)	9.451 (4.023)	7.329 (1.951)

* = Different to Air controls (* = *p* < 0.05, ** = *p* < 0.01); # = Different to ULSD (# = *p* < 0.05); a = Different to other biodiesels (*p* < 0.05).

**Table 4:** Mean (standard deviation) measurements for airway morphology and chord length and collagen % for mice exposed to Air, ULSD, Canola, or Tallow biodiesel exhaust for 2 hr/day for 8 days (*n* = 10–12 for all measurements). Pbm = perimeter of the basement membrane.

Measurement	Air	ULSD	Canola	Tallow
Total Wall Thickness (√area/Pbm)	0.123 (0.015)	0.130 (0.013)	0.128 (0.013)	0.127 (0.007)
True Wall Thickness (√area/Pbm)	0.060 (0.007)	0.057 (0.006)	0.058 (0.005)	0.058 (0.008)
Airway Smooth Muscle Thickness (√area/Pbm)	0.036 (0.003)	0.033 (0.003)	0.033 (0.003)	0.035 (0.004)
Epithelial Thickness (√area/Pbm)	0.040 (0.003)	0.039 (0.003)	0.037 (0.002)	0.038 (0.003)
Chord Length (µm)	21.07 (2.31)	21.89 (1.54)	22.42 (2.39)	23.33 (1.74) *
Collagen (%)	2.718 (0.534)	2.604 (0.230)	2.456 (0.487) a	3.262 (1.197) #a

* = Different to Air controls (* = *p* < 0.05); # = Different to ULSD (# = *p* < 0.05); a = Different to other biodiesels (*p* < 0.05).

## Data Availability

Data are available upon request.

## Supplementary Materials

The following supporting information can be downloaded at: https://www.mdpi.com/article/10.3390/ijms24065130/s1, Figure S1: Changes in gas concentration over time, Figure S2: Representative images of airway morphometry.
