# Supplementary material for: Respiratory Health Effects of In Vivo Sub-Chronic Diesel and Biodiesel Exhaust Exposure
Source: Int J Mol Sci. 2023 Mar 7;24(6):5130. doi: 10.3390/ijms24065130 (PMC10049281; doi:10.3390/ijms24065130)
Supplement: Supplementary file 1 [file ijms-24-05130-s001.zip › ijms-2256112-supplementary.pdf]

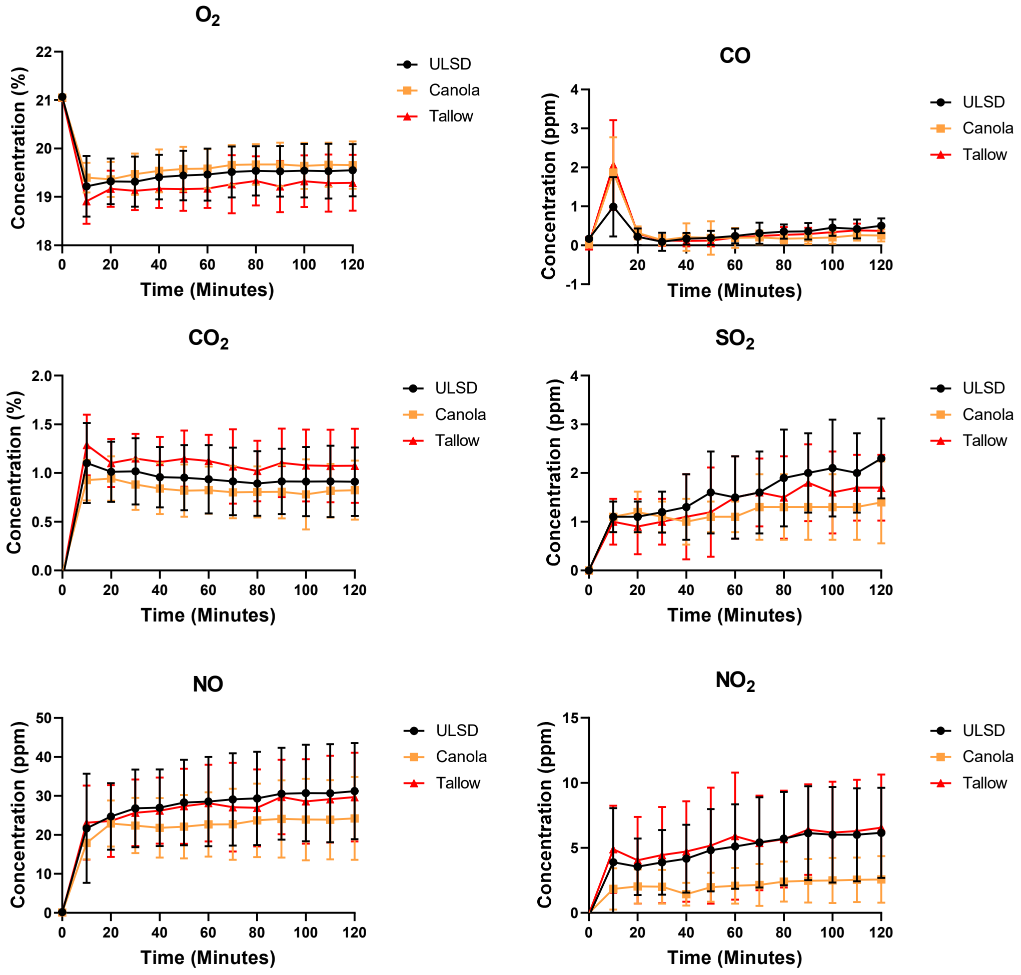

Figure S1: Changes in gas concentrations over time.

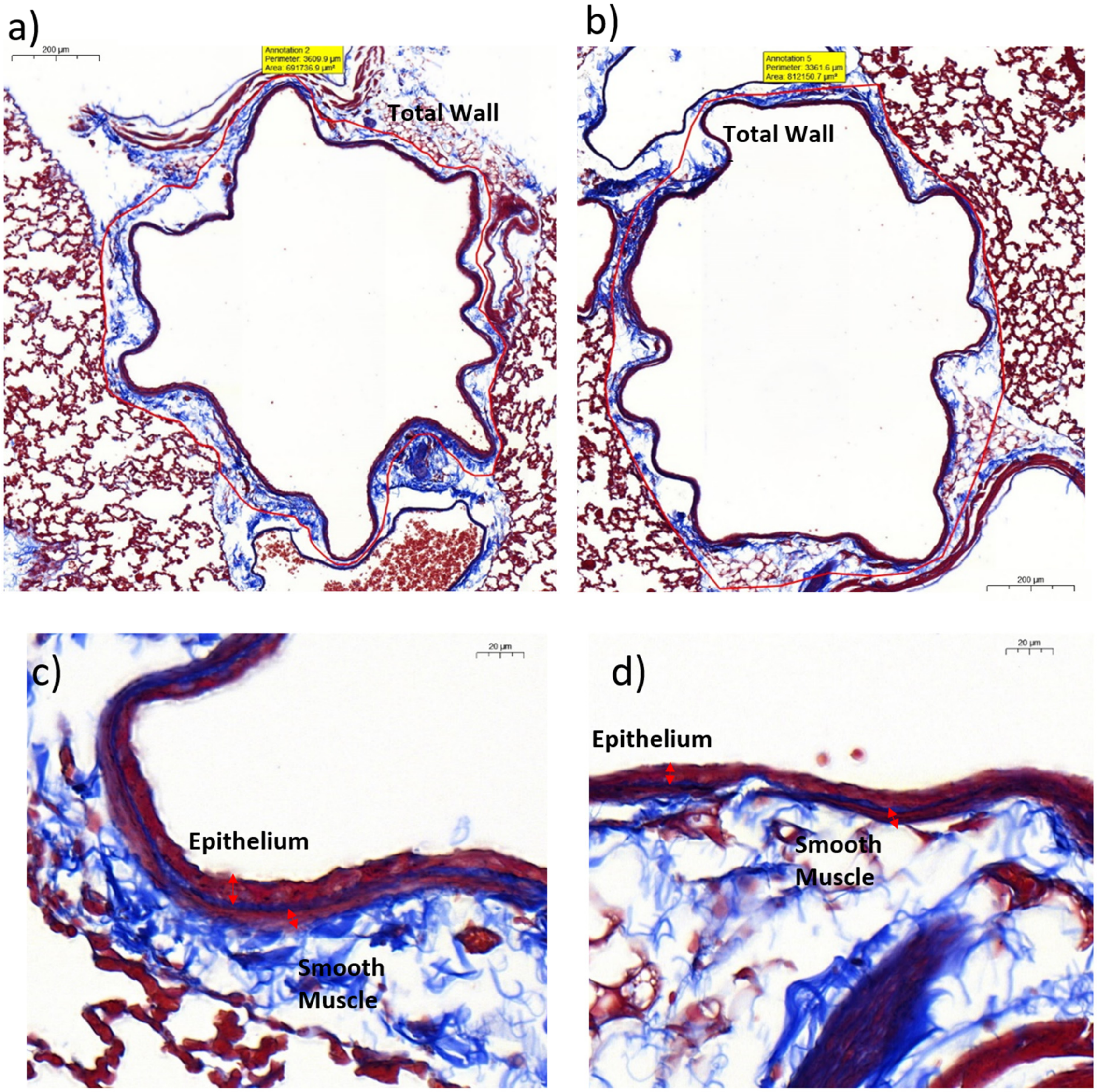

Figure S2: Representative images of airway sections stained with Masson's Trichrome. Images are taken from Air and ULSD exhaust exposed mice. (a) and (c) are whole and zoomed airway images of a representative Air exposed mouse while (b) and (d) are the same for an exhaust exposed mouse. The different airway measurements are outlined in red.
